# The Prevention and Management of COVID-19: Seeking a Practical and Timely Solution

**DOI:** 10.3390/ijerph17113986

**Published:** 2020-06-04

**Authors:** Charulata Jindal, Sandeep Kumar, Sunil Sharma, Yuk Ming Choi, Jimmy T. Efird

**Affiliations:** 1 Faculty of Science, University of Newcastle, Newcastle 2308, Australia; charujindal@gmail.com; 2 Department of Surgery, King George Medical University, Lucknow 226003, India; k_sandeep@hotmail.com; 3 Department of Medicine, West Virginia University Health Sciences Center, Morgantown, WV 26506, USA; sunil.sharma@hsc.wvu.edu; 4 Signify Health, Dallas, TX 75244, USA; yuk.ming.choi@alumni.uncg.edu; 5 Cooperative Studies Program Epidemiology Center, Health Services Research and Development (DVAHCS/Duke Affiliated Center), Durham, NC 27705, USA

**Keywords:** carnosine, COVID-19, SARS-CoV-2

## Abstract

We read with interest several manuscripts recently published in the *International Journal of Environmental Research and Public Health* (IJERPH) on the ongoing coronavirus pandemic. While these articles provide a well-rounded overview on the risk and current status of this virus, we herein add some relevant information on its etiology, prevention and management, especially for resource-limited healthcare systems. The use of protective actions is both complex and expensive. Affordable options are essential to respond to this and future viral outbreaks.

## 1. Introduction

SARS-CoV-2 (Human/Wuhan/X1) is a novel virus belonging to the genus Betacoronavirus [1,2,3]. It is believed to be of zoonotic origin, having 96% similarity at the whole genome level with other bat coronavirus samples [4]. Transmission is by way of close contact with infected individuals and animals, surface exposures, and respiratory droplets/bodily fluids, with a median of 5.1 days to start of symptoms from known contact [5]. However, person-to-person interaction remains the dominant mode for the spread of SARS-CoV-2 [6]. Approximately 100 out of every 10,000 cases will manifest symptoms after 14 days of active monitoring or quarantine [5]. The virus is associated with an acute respiratory disease called coronavirus disease 19 (COVID-19) [7]. Common symptoms of COVID-19 include fever (99%), fatigue (70%) and dry cough (59%), but in more serious cases (5%) the virus culminates in respiratory failure, septic shock, multiple organ dysfunction, and/or death [7]. Approximately 52% are asymptomatic at the time of testing, raising concerns that carriers with no symptoms could be transmitting the virus unwittingly [8,9]. A case fatality rate approaching 46% has been reported among adults between 65 and 84 years of age, especially those with comorbidities such as cardiovascular disease, diabetes, hypertension, and cancer [10]. 

COVID-19 is a member of the coronavirus family with clinical features like other coronaviruses, being associated with cognitive, enteric, hepatic and respiratory diseases. Its entry mechanism in the human body cell resembles SARS-CoV (i.e., interaction between its spike S1 protein and the N-terminal region of angiotensin-converting enzyme 2 (ACE2)) [11,12,13,14]. SARS-CoV-2 mutates rapidly and binds to ACE2 receptors present in the lungs, myocardium, and kidneys, accounting for a complex cascade of symptoms [15]. The binding affinity of SARS-CoV-2 S protein to ACE2 (a homologue of angiotensin-converting enzyme) is up to 20 times higher than its sister SARS-CoV-S protein, potentially contributing to its greater aggressiveness and transmissibility [16]. GRP78 is another receptor enabling SARS-CoV-2 to penetrate host cells by inhibiting the action of spike S1 protein [17,18].

The first case of COVID-19 was identified in Wuhan (China) in December 2019 [2,3]. Since then, approximately 6.4 million individuals have been infected globally and more than 383,000 have died [19]. By the end of January 2020, the World Health Organization (WHO) declared COVID-19 a “Public Health Emergency of International Concern (PHEIC)” and upgraded the viral outbreak to a pandemic on March 11 [20]. Owing to its often-asymptomatic presentation for days to weeks after infection, COVID-19 is increasing exponentially in densely populated and/or low-resource areas such as India, Bangladesh, Brazil, Southeast Asia, and Africa [19].

Our knowledge about the mechanism of how SARS-CoV-2 spreads inside the human body and damages lung tissues is still limited. [21] In many cases, the lungs become inflamed with excessive fluid build-up, leading to difficulties breathing [15]. Respiratory symptoms may become severe enough to require hospital administered oxygen/ventilation or may even result in complete lung failure and death [22]. Consequently, the complications associated with COVID-19 (e.g., acute respiratory distress syndrome (ARDS) and pneumonia) pose a significant burden on health care resources [15]. Even countries such as Italy and United States (USA) with a strong health care system infrastructure have struggled to control the mortality associated with COVID-19 [23,24]. Although not all viral fragments are able to replicate, fecal virus shedding may occur for up to 33 days after a negative respiratory sample, possibly exacerbating viral transmission under poor hygienic conditions [25]. These RT-PCR positive findings pose a non-negligible probability that some recovered patients still may be virus carriers. However, the virus generally cannot be isolated or cultured after day 11 of illness [10,26]. 

In this editorial, we expand on the insights offered in several recently published papers in this journal on the topic of COVID-19 [27,28,29]. This entails realizing the challenges but also implementing a practical and timely solution for managing the global health and economic impact of this virus. Confounding this effort is the large percentage of pre-symptomatic transmission which impedes the initiation of disease management measures, prompted by symptom onset of COVID-19 [9]. Additionally, there are no currently approved SARS-CoV-2 antiviral agents and the regulatory barriers to new or repurposed compounds is complex and time-consuming [30].

## 2. Epidemiology

Acute respiratory distress syndrome (ARDS) and pneumonia are the most common causes of COVID-19 associated mortality. [31] Among 191 SARS-CoV-2 positive patients, 59 died during treatment in Wuhan (China) [32]. Out of 59 patients who died, 50 had ARDS; out of 132 survivors only 4 developed ARDS. 

By mid-March 2020 in Italy, there were a total of 27,980 confirmed cases of COVID-19 with 2158 deaths [23]. The reported case fatality rate of ~7.7% was relatively higher than China (4.0%), Iran, Spain (5.7%), South Korea (0.9%), France, United States (2.4%), Switzerland (0.6%) and Germany (0.2%) [23]. The United States also has experienced a high number of infections (1,571,617 confirmed cases by the third week of May 2020), with 94,150 deaths [24]. Other countries in the Southern Hemisphere such as Brazil are reporting rapidly increasing rates of infection and deaths [33].

## 3. Laboratory Abnormalities and Clinical Features

The laboratory abnormalities associated with COVID-19 include raised levels of C-reactive protein, D-dimer, lymphocytopenia, IL-6, troponin, and CK-MB. The rise in CK-MB suggests heart injury which can be directly caused by the virus because of distribution of ACE2 receptors or due to inflammatory storm [34]. D-dimer values correlate with disease severity and are a reliable prognostic marker for in-hospital mortality in patients with COVID-19 [32]. In SARS-CoV-2 infection, dysregulation of coagulation cascades can result in worsening lung pathology [22]. Raised baseline IL-6 levels were positively correlated to maximal body temperature during hospitalization and were also associated with greater progression of CT findings [35]. Severity of COVID-19 outcomes might be associated with excessive production of pro-inflammatory cytokines and ’cytokine storm’ leading to ARDS-like disease. Therefore, therapies like IL 6 antibody blockers, stem cell therapy and transfusion convalescent plasma have been applied. Most deaths are attributable to ARDS, pneumonia and fluid leakage into the lungs [6]. Lymphocytopenia also is a prominent feature of COVID 19. Autopsy findings have revealed that secondary lymphoid tissues have been destroyed yielding splenic atrophy in these patients [36].

Among the plethora of symptoms and signs caused by SARS-CoV-2, there is a marked variation in its presentation and severity and even in different strata of the same population. These symptoms have been known to develop within an incubation period of 14 days which can vary from 3 to 28 days [37]. While the most common symptoms encountered include fever, dry cough, dyspnea, and breathlessness, a wide range of other nondescript symptoms have been reported (e.g., aches and pains, sore throat, diarrhea, conjunctivitis, headache, loss of taste and sense of smell, rash on skin, and some discoloration of fingers and toes) [7]. Serious symptoms such as chest pain or pressure, loss of speech or movement also encompasses its clinical vignette [15]. Most patients have mild to moderate symptoms, but the disease can cause severe medical complications—especially among the elderly, immuno-compromised and those with co-morbidity such as diabetes and hypertension [10].

Another prominent clinical feature is endothelium damage—a mimicry of vasculitis. Gangrene in extremities and bowel have been reported. Direct endothelial injury by the virus leading to disseminated intravascular coagulation (DIC) has been reported [34]. This is evident by congested blood vessels especially in the alveolar septum, small vessels showing hyperplasia, vessel wall thickening, luminal stenosis, occlusion, and focal hemorrhage in the vascular system. The cardiovascular system shows arrhythmias (atrial fibrillation, ventricular tachyarrhythmia and fibrillation), fulminant myocarditis, thromboembolism and DIC [34]. Pulmonary imaging studies reveal ground glass opacities, consolidation, crazy paving, cavitation, discrete nodules, pleural effusion, and lymphadenopathy [38].

## 4. Risk Mitigation

According to the Centers for Disease Control, there are two ways to control the damage associated with a viral infection: (1) reduce the spread of virus and, (2) decrease the associated disease severity [39]. Common precautions such as maintaining proper distance with sick people (at least 2.5 square meters), staying at home if sick, covering face while coughing and sneezing in public, and frequently washing hands are helpful in reducing the spread of the virus [6]. Most countries throughout the world have issued social distancing and hygiene associated notices, with some locales mandating a complete lockdown to slow the transmission of SARS-CoV-2 [40]. Contact tracing for those infected and quarantined remains an astute practice [9].

Although there is a paucity of data supporting their use and safety, various pharmacologic agents/classes (e.g., therapeutic antibodies, cytokines, and nucleic acid-based therapies targeting virus gene expression) have been proposed to extenuate the severity of COVID-19 symptoms. Compounds receiving recent attention in the literature include (1) Hydroxychloroquine/chloroquine, (2) Oseltamivir, (3) Remdesivir/Ribavirin/Favipiravir, (4) ACE2, (5) Tocilizumab/Sarilmab, (6) Lopinavir-ritonavir/Darunavir, (7) Baraticinib, (8) non-steroidal anti-inflammatory drugs, and (9) Glucocorticoids, (10) anti H1 compounds (Cetirizine, Desloratadine, Levocetirizine), (11) Arbidol, and (12) Camostat mesylate [27,30,41,42,43]. Virus-induced host immune system response and proposed targets for some of the above repurposed compounds encompass (1) targeting S protein/ACE2; inhibiting membrane fusion of the viral envelope; binding to host cell receptor ACE2 (Arbidol), (2) inhibiting TMPRSS2; preventing viral cell entry; priming S protein to facilitate its binding to ACE2 (Camostat mesylate; (3) inhibiting viral entry and endocytosis; supporting host immunodulatory effects (Chloroquine/Hydroxychloroquine), (4) binding IL-6 receptor, preventing IL-6 receptor activation; inhibiting IL-6 signaling (Tocilizumab/Sarilumab), (5) inhibiting 3-chymotrypsin-like protease; proteolysis of viral polyprotein into functional units (Lopinavir/Darunavir), (6) inhibiting viral RdRp (Ribavirin/Remdesivir/Favipiravir), and regulating blood pressure and volume of the cardiovascular system by targeting angiotensin AT2 receptor (L-163,491) [30,42]. Many of these agents are being used anecdotally based on *in vitro* extrapolation or retrospective registry analysis, but caution is warranted in their unproven (or pilot) application in humans. Specific agents such as glucocorticoids, JAK inhibitors (baricitunib, ruxolitinib), and tocilizumab may have undesirable long-term side effects. For example, steroids and JAK inhibitors may blunt the interferon alpha (*IFN*-α) response that is supposed to be beneficial against the virus [44].

Analyzing observational data from 8910 patients hospitalized with COVID-19 at 169 facilities in Asia, Europe, and North America (Surgical Outcomes Collaborative) between December 20, 2019 and March 15, 2020, the use of ACE inhibitors was associated with a lower occurrence of in-hospital deaths (odds ratio = 033, 95%CI = 0.20–0.54) [45]. This result was independent of the patient’s age, sex, coronary disease, congestive heart failure, arrhythmia, COPD, smoking status, and use of ARBs or statins. However, it is important to note that this was not a randomized controlled trial and confounding factors not accounted for in their analysis may have biased results. Additionally, a primary hypothesis was not pre-specified, and results were not adjusted for multiple testing. In a larger covariate-adjusted analysis (by the same team) of 96,032 COVID-19 registry patients, the uses of hydroxychloroquine (hazard ratio = 1.34, 95%CI = 1.22–1.46), hydroxychloroquine with a macrolide (1.45, 1.37–1.53), chloroquine (1.37, 1.22–1.53), and chloroquine with a macrolide (1.37, 1.27–1.47) were each independently associated with an increased risk of in-hospital mortality, in contrast to the above-mentioned use of ACE inhibitors [46]. An increased risk of *de novo* ventricular arrhythmia during hospitalization also was associated with these drug combinations. 

Using untargeted computational modeling of molecular binding structures, the histamine-2 receptor antagonist famotidine has been identified for its propensity to inhibit 3-chymotrypsin-like protease (3CL^pro^), which process proteins important for viral replication [47]. In a study of 1620 patients who tested positive for SAR-CoV-2 within 72 hours following admission (and not initially intubated), the use of famotidine was significantly associated with improved survival/intubation times, adjusting for baseline characteristics (hazard ratio = 0.42, 95%CI = 0.21–0.85) [48]. In contrast, a protective effect was not observed for the use of proton pump inhibitors in this study. While intriguing, this was a single center study and additional randomized clinical trials are needed to replicate the findings in the general COVID-19 population.

Preliminary evidence from a randomized clinical trial of 1059 patients suggests a possible benefit for the investigational agent remdesivir (GS-5734), an inhibitor of viral RNA-dependent, RNA polymerase (with viral suppression previously shown against SARSCoV and MERS-CoV) [20]. Those who received remdesivir had a shorter recovery time (median of 11 days versus 15 days for referents; P < 0.0001) in adults hospitalized with COVID-19. Treated patients also manifested lower rates of acute respiratory failure and viral pneumonia. Under an emergency-use authorization, the U.S. Food and Drug Administration (FDA) has authorized the use of remdesivir for adults and children with severe COVID-19 disease. In summary discussion, the authors recommend that “future strategies should evaluate antiviral agents in combination with other therapeutic approaches or combinations of antiviral agents to continue to improve patient outcomes.”

**Figure 1:**
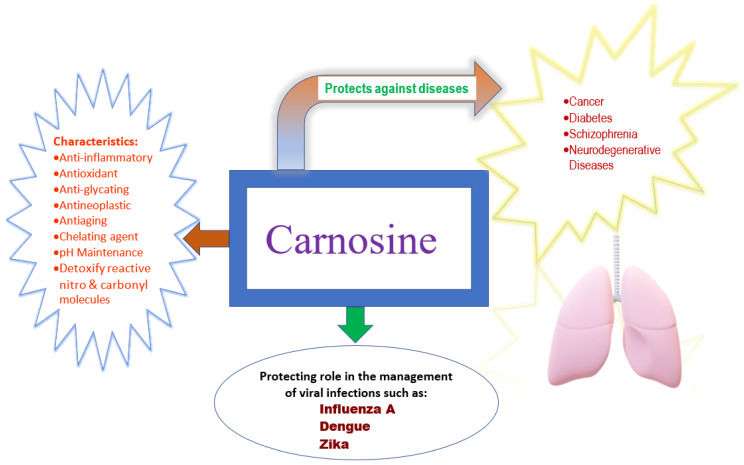
Characteristics and possible therapeutic potential of carnosine.

Below, we mention the properties of carnosine, a non-pharmacologic agent with antiviral activity and other therapeutic functions (antioxidant, antiglycation, metal ion chelator, immune response modulatory; see Figure 1) [49,50]. In response to the above-mentioned recommendation regarding the combination of therapeutic approaches, carnosine shows promise as a low-cost stopgap supplement, with potential additional benefits in the management of COVID-19 (especially until a vaccine becomes available or while putative pharmacologic agents undergo additional confirmatory testing). While there currently are no conclusive clinical trial data in support of any prophylactic therapy, supplemental compounds like carnosine are worthy of investigation [42]. This is particularly salient given the purported benefit of carnosine in mitigating comorbidities associated with COVID-19. 

## 5. Carnosine

Carnosine (*N*-b-alanyl-L-histidine), an effective antiviral, antioxidant and antiglycating agent, is a naturally occurring dipeptide [51,52,53,54,55]. It is present in skeletal system, cardiac muscles and brain of vertebrates [56,57]. This multifaceted compound also exhibits anti-inflammatory, anti-aging, antihypertensive, and antineoplastic properties, and is helpful in removing carbonyl species from the human body and maintaining its pH [50,57,58]. Carnosine acts as a chelating agent to reduce levels of heavy metals in the bloodstream [59]. A formulation of Zinc (which has intrinsic antiviral activity to the family of coronaviruses) and L-carnosine interacts synergistically to suppress inflammatory processes associated with a host of debilitating diseases [60,61]. Carnosine is available as a low-cost, non-prescription supplement and may have benefits in the global management of COVID-19 and related viruses that may emerge in the future.

A common feature of all virus-induced human diseases is the sustained increase in levels of iNOS and Nitric Oxide (NO) [62,63]. While increased NO concentrations are protective against microbial infections, the opposite is true in the case of viral infections [64]. In the latter scenario, NO reacts with oxygen free radicals to produce highly reactive peroxynitrites, which in turn damage tissue and DNA through the nitrosylation of cellular proteins and molecules [65,66,67]. Owing to its anti-inflammatory and antioxidant properties, carnosine can reduce the concentration of highly reactive peroxynitrites in the human body, aiding the immune fight against viral infections such as influenza A, dengue fever and Zika [68]. Furthermore, in liver cell culture assays, carnosine has been shown to significantly inhibit viral genome replication and to ameliorate cell viability post infection [69].

In a laboratory study of BALB/c female mice infected with H9N2 influenza virus, 7 consecutive days of carnosine administered orally (10 mg/kg body mass) significantly reduced levels of TNF-a, IL-1b, TLR-4 mRNA and protein, as well as decreasing overall mortality (43% versus 75%, P < 0.05). An improvement in pathological lung lesions, decreased lung wet mass ratio, and reduced myeloperoxidase activity also was reported. We similarly hypothesize that the oral administration of carnosine may play an important role in reducing the lung tissue damage associated with SARS-CoV-2 infection and hence associated morbidity and mortality [70,71].

Genetic variants in Apolipoprotein E (ApoE), which are involved in regulatory checkpoint processes of the innate immune system and associated antigen–antibody complexes, also may underlie the therapeutic effects of carnosine, autonomous of its antiviral properties [72,73,74]. Compared with ApoE e3e3 homozygotes, COVID-19 positivity occurs more frequently among e4e4 homozygotes (OR = 2.3, 95%CI = 1.7–3.2), with increased severity being independent of pre-existing dementia, cardiovascular disease, and type-2 diabetes [75]. Both ACE2 and ApoE are highly co-expressed genes in type II alveolar cells in the lungs [76]. Among ApoE4 positive carriers, carnosine supplementation conveys positive benefits for mild cognitive impairment and blood flow in the prefrontal cortex of the brain [77,78]. Dietary administration of carnosine also has been shown to prevent early atherosclerotic lesion formation in ApoE-null mice, as well as attenuating renal disease [79,80].

The regular administration of carnosine (in combination with forskolin, homotaurine, vitamins B1, B2, and B6, folic acid, and magnesium) for 2–4 months, has been shown to be safe for humans of different age groups. In two separate studies, one in obese patients with Type-II diabetes and the other in Gulf-War Veterans, the use of carnosine for 2 weeks was well tolerated without any reported adverse reactions. While carnosine holds promise as a useful therapeutic tool, the overall safety of this supplement in patients with COVID-19, many of whom have comorbid conditions such as cancer, heart disease, and diabetes, has yet to be tested [49,68,81,82,83]. Information pertaining to the oral bioavailability and bioaccessibility of carnosine in human cohort studies also is limited at this time [84]. Accordingly, clinicians are advised to use prudent judgement when recommending the use of this (or any other over the counter compound) for their patients with COVID-19.

## 6. Conclusions

There is an old parable that “you cannot remove all stones form the road, it’s better to wear shoes”. It is applicable here too. Containing COVID-19 is encumbered by the high rate of viral transmission in infected patients, as well as its asymptomatic period of infection [26,85]. Nonetheless, given the promising results observed for other virus-related human and animal diseases, we are optimistic that carnosine may be helpful in reducing the severity of COVID-19 symptoms and associated comorbidities.

Vaccination is the optimal means to control the morbidity and mortality associated with viral infection. However, developing a vaccine for a novel virus (with the potential to mutate) is a lengthy process and may take 1–2 years or longer to fully establish safety and efficacy. The administration of carnosine to patients with COVID-19 during this interval may be helpful in curtailing this disease and merits future study. Its use also may prove beneficial in offsetting the associated burden on health care systems in resource-poor countries, which struggle with economic inequalities and the high cost and delivery of medical care [86]. 

To date, the prevention and treatment of COVID-19 remains a challenge, with few definitive options. This is particularly concerning given that RT-PCR results may remain positive to Week 6 among infected individuals [87]. Patients are advised to seek medical consultation at the first signs of infection. They also should adhere to standard supportive care (e.g., symptomatic outpatient management, full intensive care support) under the supervision of a licensed medical practitioner when diagnosed with COVID-19 or related viral infections [42].
